# Systemic immunotherapy delays photoreceptor cell loss and prevents vascular pathology in Royal College of Surgeons rats

**Published:** 2012-09-06

**Authors:** Grazyna Adamus, Shaomei Wang, Madison Kyger, Aneta Worley, Bin Lu, Gregory G. Burrows

**Affiliations:** 1 Ocular Immunology Laboratory, Casey Eye Institute, Oregon Health & Science University, Portland, OR; 2 Tykeson MS Research Laboratory, Department of Neurology, Oregon Health & Science University, Portland, OR; 3 Department of Biochemistry and Molecular Biology, Oregon Health & Science University, Portland, OR

## Abstract

**Purpose:**

Degenerative retinopathies, including retinitis pigmentosa, age-related retinal degeneration, autoimmune retinopathy, and related diseases affect millions of people around the world. Currently, there is no effective treatment for most of those diseases. We investigated systemic recombinant T-cell receptor ligand (RTL) immunotherapy for preventing retinal degeneration and vascular damage in the Royal College of Surgeons (RCS) rat model of retinal degeneration.

**Methods:**

RCS rats were treated with RTL220 tethered to interphotoreceptor retinoid binding protein (IRBP) peptide or control RTL101 without peptide by subcutaneous administration starting at the onset of photoreceptor degeneration or after the degenerative process began daily or every other day and performed for a 13-week period. The retinal cross sections and whole mounts were prepared to determine histopathology, leaking vessels, and formation of vascular complexes. Immunofluorescent studies evaluated microglia and monocyte chemoattractant protein-1 chemokine in treated retinas. Optokinetic studies were performed to determine visual acuity.

**Results:**

Systemic treatment with RTL220 prevented decreases in outer nuclear layer (ONL) thickness and showed a significantly higher number of nuclei than control rats treated with RTL101 or vehicle. RTL220 was also effective in protecting retinal vasculature from leakage and the formation of abnormal vascular complexes even when the treatment was administered after the degenerative process was initiated. Visual acuity measurement showed that rats treated with RTL220 performed significantly better than those with RTL101 and untreated age-matched controls at P60 and P90. Biodistribution studies showed that RTL220 cleared slowly from the administration site. Moreover, RTL220-treated retinas had a significantly reduced number of activated microglia in the subretinal space, decreased monocyte chemoattractant protein-1 production in the retina, inhibited T-cell responses, and reduced anti-interphotoreceptor retinoid binding protein autoantibody titers. Treatment with the control RTL101 (without a specific peptide tethered) or vehicle alone did not inhibit microglia activation or protect photoreceptors or vasculature.

**Conclusions:**

RTL therapy augmented photoreceptor cell survival, protected vasculature, and increased visual function in the RTL rat. Targeting chronic autoimmunity with RTLs can be an effective therapeutic alternative in delaying retinal degeneration. Subcutaneous delivery of RTLs alone or combined with other drugs could be an attractive option for long-term therapy for retinal degenerative diseases.

## Introduction

Degenerative retinopathies, including retinitis pigmentosa (RP), age-related retinal degeneration (AMD), autoimmune retinopathy, and related diseases affect millions of people around the world. During retinal degeneration, loss of photoreceptor cells results in secondary vascular leakage and migration of the retinal pigment epithelium (RPE) into blood vessels leading to severe vascular dysfunction [1-4]. Such changes exacerbate the deterioration of vision in these patient groups, leading to blindness. Treatment options for these conditions are limited. Although the mechanisms leading to development of degenerative diseases remain largely unknown and appear to be complex, they are likely caused by a combination of factors, including inflammatory factors [5-9]. Recent studies have shown that innate and adaptive immune responses play a critical role in the pathogenesis of several different neurodegenerative diseases, including AMD [7,10]. In retinal diseases, there is evidence of only mild chronic inflammation with the presence a few vitreous cells, aqueous flare, and in some cases cystoid macular edema. There is also evidence of antiretinal autoantibodies, cytokines, and the increased presence of various leukocytes, including macrophages, CD4^+^ T cells, CD8^+^ T cells, and activated T-suppressor cells, in the circulation and in eyes with various forms of retinal degeneration, while normal controls demonstrate only rare macrophages [5,7,8,11,12]. The pathology of AMD lesions demonstrate signs of persistent chronic inflammatory damage, including not only mild infiltration of macrophages and accumulation of microglia but also the presence of inflammatory components such as complement factors and proinflammatory cytokines/chemokines in the drusens [5,13]. The build-up of toxic waste products in the retina causes progressive loss of vision. As an effect, microglia, activated by photoreceptor death, kill adjacent cells. All those findings provide a rationale for immunotherapy, including T cell–based therapy specific for antigens derived from retinal proteins that we have explored in our studies. Our goal was to treat retinal degenerative diseases without performing an intraocular injection that could disrupt an already fragile retina. Since most of these diseases progress slowly, long-term treatment is needed, and tolerization using recombinant T-cell receptor ligands (RTLs) could offer such an approach.

The photoreceptor degeneration found in several human diseases shows features similar to those observed in animals with inherited retinal degeneration, including the Royal College of Surgeons (RCS) rat model of retinal degeneration and vascular pathology used in our studies. Some histopathological features observed in the RCS rat are similar to those seen in certain forms of RP, including the loss of photoreceptors, invasion of RPE cells into the inner retina, narrowing of blood vessels and thickening of the vascular basal lamina, the presence of neovascular formations, “leaky” blood vessels, and the formation of preretinal membranes [14]. These rats have a mutation in the gene for the Mer receptor tyrosine kinase (MerTK) that results in dysfunction of RPE cells leading to progressive loss of rods and cone cells over time [15]. RTLs ameliorate experimental autoimmune uveitis [16,17] and autoimmune optic neuritis [18], but the effect on spontaneous retinal degeneration that is not acute inflammatory has not been investigated until now. In our studies, we evaluated RTL immunotherapy to prevent retinal and vasculature degeneration in the RCS rat model. Our results revealed that RCS rats treated with recombinant T-cell receptor ligand 220 (RTL220) bearing a retina-specific peptide interphotoreceptor retinoid binding protein (IRBP) peptide at the onset of photoreceptor degeneration or when the degenerative process started preserved the retina and significantly slowed the progression of retinal vasculature from degeneration.

## Methods

### Animals

Dystrophic Royal College of Surgeons (RCS) rats were bred and housed at the Oregon Health Sciences University Animal Care Facility according to institutional and federal guidelines. Both genders were used for experiments in groups of three to six when they reached postnatal age of 21 (P21) or 30 (P30) days. All animal experimentation procedures adhered to the Association for Research in Vision and Ophthalmology (ARVO) Resolution on the Use of Animals in Research and have been approved by the Oregon Health Sciences University animal committee.

### RTL220 treatment

RTL220 is a biologic consisting of the membrane distal β1 and α1 domains of RT1.B rat class II major histocompatibility complex (MHC) molecules bearing N-terminal covalently linked IRBP_1177–1191_ antigenic peptide (ADGSSWEGVGVVPDV). Controls included RTL101 (“empty” RTL) consisting of the β1α1 MHC molecule without an antigenic peptide tethered, the IRBP_1177–1191_ peptide alone, or vehicle (saline). All RTLs were produced in *Escherichia coli* and chromatographically purified as follows [16,19]. *Escherichia coli* strain BL21(DE3) cells were transformed with the pET21d+/RTL (‘empty”) and pET21d+/RTL/peptide vectors and were grown in 1 l cultures to mid-logarithmic phase (OD 600=0.6–0.8) in Luria-Bertani broth containing carbenicillin (50 μg/ml) at 37 °C. Recombinant RTL production was induced by addition of 0.5 mM isopropyl β-D-thiogalactoside (IPTG). After incubation for 3 h, the cells were centrifuged and stored at –80 °C before processing. The cell pellets were resuspended in ice-cold phosphate buffed saline (PBS), pH 7.4, and sonicated for 4×20 s with the cell suspension cooled in a salt/ice/water bath. The cell suspension was then centrifuged, then the cell pellet was resuspended and washed three times in PBS and finally in 20 mM ethanolamine/6 M urea, pH 10, for 4 h. After centrifugation, the supernatant containing the solubilized recombinant protein was collected, purified and concentrated by FPLC ion-exchange chromatography using Source 30Q anion-exchange media (Pharmacia Biotech, Piscataway, NJ) in an XK26/20 column (Pharmacia Biotech), using a step gradient with 20 mM ethanolamine/6 M urea/1 M NaCl, pH 10. After purification, the protein was dialyzed against 20 mM ethanolamine, pH 10.0, which removed the urea and allowed refolding of the recombinant protein correctly. Next, RTLs were dialyzed into PBS and concentrated by centrifugal ultrafiltration with Centricon-10 membranes (Amicon, Beverly, MA). Finally RTL was purified to homogeneity using size exclusion chromatography on Superdex 75 media (Pharmacia Biotech) in HR16/50 column (Pharmacia Biotech).

In Regimen 1, randomized groups of female and male RCS rats at P21 were treated with subcutaneous injection (s.c.) at the back of the rat with 20–200 µg RTL220, or RTL101 per dose every other day for 10 days and once a week thereafter until P90. The rats were examined at P60 and P90. In Regimen 2, randomized groups of female and male RCS rats at P30 received daily s.c. administration of 20 µg RTL220 per dose every other day until P90. Controls consisted of the following groups: RTL101, vehicle, or free IRBP_1177–1191_ peptide in saline (the same peptide that is a part of RTL220), and untreated age-matched rats (n=6/group). At the end of the experiments, the eyes were removed and immersed in 4% paraformaldehyde for 1 h and then in 30% sucrose overnight, followed by freezing in optimal cutting temperature (OCT) compound (Tissue-Tek OCT compound, Sakura Finetek, Torrance, CA) at −80 °C. Cryosections (10 µm) were stained in hematoxylin/eosin or crystal violet to visualize the outer nuclear layer and to assess histopathology and retinal lamination. The images were taken using an Olympus BH-2 light microscope and Olympus DP21 camera at 10× and 20× magnifications. The total number of preserved nuclei in the outer nuclear layer (ONL) was measured in histological sections of the retina in 1 mm^2^ areas at 500-µm intervals throughout the retina, and the number of nuclei was recorded at 0.5, 1.0, 1.5, 2.0, 2.5, and 3 mm from the optic disc from each treatment group from nasal to temporal direction, including the optic disc region. Six retinas were counted per experiment by a blinded observer, and an average count of nuclei in the ONL was obtained. ONL density was expressed as mean±standard error of the mean (SEM). The Mann–Whitney nonparametric test and the one-way ANOVA (ANOVA) nonparametric test were used to generate p values. The results were graphed using Prism.

### Biodistribution studies

RTL220 protein was labeled to near-infrared fluorescent IRDye800CW N-hydroxysuccinimidyl ester (NHS; LI-COR, Lincoln, NB) according to the manufacturer’s recommendations for small proteins using a stock concentration of IRDye800CW at 1 µg/ml and RTL220 at 1 mg/ml in PBS. Unbound dye was separated from the conjugate on the column. Intact RCS rats (n=7) injected with fluorescent RTL220 were imaged (774/789 nm) in real time at 0, 10, 30, 60, and 120 min and then once a day using an optical imaging Odyssey Sa Infrared Imaging System (LI-COR) under CO_2_ anesthesia (2% isoflurane anesthesia by inhalation). The advantage of using this dye is that it is stable for in vivo use and has low background autofluorescence with low light scattering. The experiments were repeated 3 times per dose and low variance was observed (SEM within 5%) between individual rats. The data are presented as integrated intensity and plotted using Prism, showing two groups of individual rats presented in the picture that received a single or triple dose of fluorescent RTL220. The data are presented as integrated intensity and graphed using GraphPad Prism.

### Enzyme-linked immunosorbent assay

Enzyme-linked immunosorbent assay (ELISA) polystyrene plates were coated with 1 µg IRBP_1177–1191_ peptide per well in 0.1 M Tris–HCl buffer, pH 9.0 (coating buffer) overnight at room temperature. After the wells were washed with the coating buffer and blocked with 1% BSA (BSA) in PBS for 1 h, diluted serum at 1:100 was added to each well, allowed to incubate for 1 h, and then washed. Next, the wells were incubated 1 h with 1:2000 diluted biotinylated antirat immunoglobulin G (IgG; H and L chain, Invitrogen, Grand Island, NY) antibodies followed by 30-min incubation with streptavidin conjugated to peroxidase (1:5,000; Invitrogen). Color reaction was developed for 30 min by incubation with peroxidase substrate (2,20-azino-bis-(3-ethylbenz-thiazoline-6-sulfonic acid)) in 0.1 M citrate–phosphate buffer, pH 4.5, containing 3% H_2_O_2_, and immediately measured at 415 nm using a Bio-Rad Microplate Reader (Bio-Rad Laboratories, Hercules, CA).

### Lymphocyte proliferation assay

The assay was performed in 96-well tissue culture plates in triplicate using RPMI medium containing 10% FBS, 5×10^−5^ M 2-mercaptoethanol, and 50 μg/ml gentamicin. Splenocytes were seeded at a density of 2×10^5^ per well and incubated with RPMI medium only, 1 μg concanavalin A, 5 μg IRBP_1177–1191_ peptide at 37 °C in 5% CO_2_ for 72 h, and then pulsed with 1 μCi tritiated thymidine/well for an additional 18 h. The cells were harvested onto a glass fiber filter, and the thymidine uptake was assessed with the liquid scintillation counting in a Betaplate counter (Wallac Pharmacia, Finland, model 1250; Espoo, Finland). The data were expressed as a stimulation index, which was calculated by dividing the proliferation (cpm incorporated) measured in the presence of antigen by the proliferation measured with medium alone.

### Fluorescein isothiocyanate angiography

Rats received 50 mg/ml of fluorescein isothiocyanate–dextran 70000 (FD70S; Sigma, St. Louis, MO) in PBS intravenously 30 min before euthanasia with 100% CO_2_ overdose by inhalation. Then the eyes were dissected. The dorsal side of each right eye was marked before enucleation. The cornea was punctured before fixation before being suspended in 4% paraformaldehyde for 30 min at room temperature and kept away from light. Retinas were removed from the eyes under a dissecting microscope, and retinal whole mounts were made by four radial cuts representing the dorsal, ventral, temporal, and nasal sides. Then the retinas were mounted on glass slides. Next, consecutive retinal images from the left to right were captured at 10× magnification with a Leica DM5000B (Leica Microsystems Buffalo Grove, IL) fluorescent microscope, and the images were merged using Microsoft ICE software (Microsoft Corporation, Seattle, WA) to obtain the whole image of the retina. The leaking vessels were counted from the entire retinal mounts (n=6/group) and are presented as a bar graph.

### Nicotinamide adenine dinucleotide phosphate–diaphorase staining

After fluorescent images were captured, the same retinal whole mount was carefully transferred into a vial with 2 ml PBS and washed for 30 min with gentle shaking. The retinas were then transferred to a new vial containing 2 ml of 0.02% beta-nicotinamide adenine dinucleotide phosphate (NADPH)-reduced tetrasodium salt (MP Biomedicals, Solon, OH), 0.04% nitroblue tetrazolium chloride (Fisher Scientific, Pittsburgh, PA), and 3% Triton X-100 (Sigma) in PBS and incubated on a shaker for 2 h at room temperature. Next, the retinas were gently washed with PBS for 30 min and mounted on glass slides, dehydrated with alcohol, and covered with DPX Mounting Medium (Leica Microsoft, Buffalo Grove, IL). Consecutive images of the stained retinal sections were taken using an Olympus BH-2 light microscope (Olympus America, Inc.) and Olympus DP21 camera at 10× magnification, and then the images were merged using Microsoft ICE software to obtain the whole retinal image. Abnormal vascular complexes were counted in the entire retina (n=6/group) and presented as a bar graph.

### Spatial visual acuity

The RCS rats were tested for spatial visual acuity at P60 and P90 post-RTL treatment using an Optometry testing apparatus (CerebraMechanics, Lethbridge, Canada) that contained a rotating cylinder displaying a vertical sine wave grating presented in virtual three-dimensional space on four computer monitors arranged in a square [20]. This noninvasive method uses the optokinetic tracking response to access functional vision using the virtual optokinetic system by optokinetic stimulation [21]. Unrestrained RCS rats were placed on a platform in the center, where they tracked the grating with reflexive head movements as described previously [22]. A unrestrained rat was placed on a platform positioned in the center of an arena created by a quad-square of computer monitors. Optokinetic stimulation produced by rotating large drums around an animal. A virtual cylinder was calculated by a computer in a three-dimensional coordinate system and projected onto the four monitors. In each trial, we judged whether the rat made slow tracking movements with its head and body in the direction of rotation. The spatial frequency, contrast, and direction of rotation of the grating on the cylinder was changed in real-time. The moving, full-field stimulus invokes slow eye and head movements. The acuity threshold was quantified by increasing the spatial frequency of the grating using a psychophysics staircase progression until the following response was lost, thereby defining the acuity.

### Fluorescent immunohistochemistry

Ten-micron rat eye cryosections were post-fixed with 4% paraformaldehyde for 10 min followed by blocking with 10% normal goat serum with 1% BSA in PBS for 60 min. Next, the blocked sections were incubated with specific primary antibodies overnight at 4 °C as follows: anti-Iba1 (1:1,000; Abcam, Cambridge, MA), anti-monocyte chemotectic protein-1 (MCP-1; 1:60; PeproTech, Rocky Hill, NJ), and anti-rhodopsin mAb R2–12N (our own, 1:2,000). After washing, appropriate fluorescent secondary antibodies conjugated to Alexa Fluor 488 (1:1,000; Invitrogen) or biotin-labeled antirat IgG (1:2,000; Invitrogen) followed by Texas Red labeled to streptavidin (1:2,000; Vector Laboratories, Inc. Burlingame, CA) were added for 1 h. The sections were washed in PBS, and then the mounting reagent, which inhibited fluorescence quenching and contained 4',6-diamidino-2-phenylindole for nuclear staining, was added to seal the sections. The immunofluorescent labeling was evaluated using an Olympus Fluoview 1000 confocal microscope, and pseudocolor images were acquired for analysis. A negative control contained secondary antibodies only.

### Statistical analyses

Statistical analysis was performed with Prism GraphPad software using a two-tailed Student *t* test, or the significance between the controls and treatment groups was determined with one-way ANOVA (ANOVA). Differences with a p value <0.05 were considered significant and are denoted with an asterisk.

## Results

### RTL220 protects photoreceptors and its function

**Figure 1:**
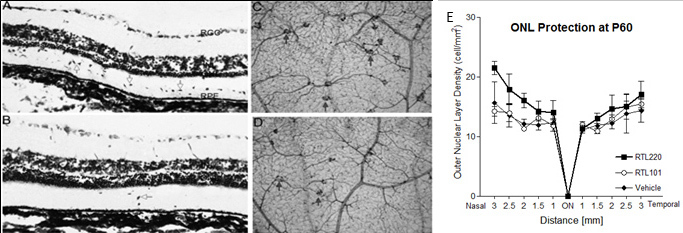
Retinal protection by RTL220 therapy. RTL220 therapy protects RCS retina from degeneration. **A**, **B**: Increased number of the outer nuclear layer rows was observed after RTL220 treatment (**B**) compared to RTL101 treatment (**A**) at P60 with RTL220 (200 μg/dose) treatment stared at P21 (the onset of degeneration); the retinas were stained with cresol violet. Arrows point at migrating cells to the outer retina (ganglion cell layer [GCL], retinal pigment epithelium [RPE]). **C**, **D**: Representative vascular pathology is shown for retinal whole mounts collected at P90 and stained with nicotinamide adenine dinucleotide phosphate (NADPH)-diaphorase to identify vascular complexes (arrows). Photomicrographs are showing the vascular complexes from middle part of a control retina (**C**) and dramatically reduced in RTL220-treated rats (**D**). **E**: Outer nuclear layer (ONL) protection was present at P60 with RTL220 (100 μg/dose) treatment stared at P30 (degeneration has begun). Graphs represent nuclei density counts from RTL treatments started at P30 and performed till P60 treated with RTL220; measurements of the ONL density at cells/mm^2^ were determined from histological sections from each treatment group and were recorded at 0.5, 1.0, 1.5, 2.0, 2.5, and 3 mm from the optic disc region. The error bars represent SEM (n=6; p=0.041).

The RCS rats displayed a progressing decrease in the ONL thickness due to the loss of photoreceptors over a 90-day period. We hypothesized that an anti-inflammatory agent such as RTL220 would slow the progression of degeneration if immunological factors contribute to pathogenicity of retinopathy in those rats. To evaluate the effectiveness of the RTL therapy, we selected two starting time points to administer the RTL treatments: (i) at the onset of degeneration at P21 or (ii) when degeneration began at P30. Then the rats were examined on P60 (approximately four layers of nuclei in the untreated ONL) and P90 (one layer of nuclei in the untreated ONL left). In the first trial, the RCS rats received a subcutaneous injection starting at P21 of 200 µg RTL220 and RTL101 (lacking peptide Ag) every other day for 10 days and then once a week until P90. Figure 1A,B shows that at P60 a few layers were present in the ONL in the control RCS rats, but applying RTL220 dramatically delayed photoreceptor degeneration. RTL220-treated retinas had five to six layers of nuclei of photoreceptors in the ONL whereas the RTL101-treated retina had only three to four layers left, indicating extensive preservation of photoreceptors in the RTL220-treated retinas compared to the RTL101-treated rats. Moreover, when the rats treated using the same protocol were examined at P90, they also showed a significant reduction in the formation of pathological vascular complexes (Figure 1C,D).When 200 μg of RTL220 was administered at P30 using the same protocol the ONL was also significantly protected (Figure 1E). The overall count of the remaining photoreceptor nuclei showed a significant difference between the controls and the RTL220-treated retinas (n=6/group, p<0.05), indicating that RTL220 inhibited degeneration.

**Figure 2:**
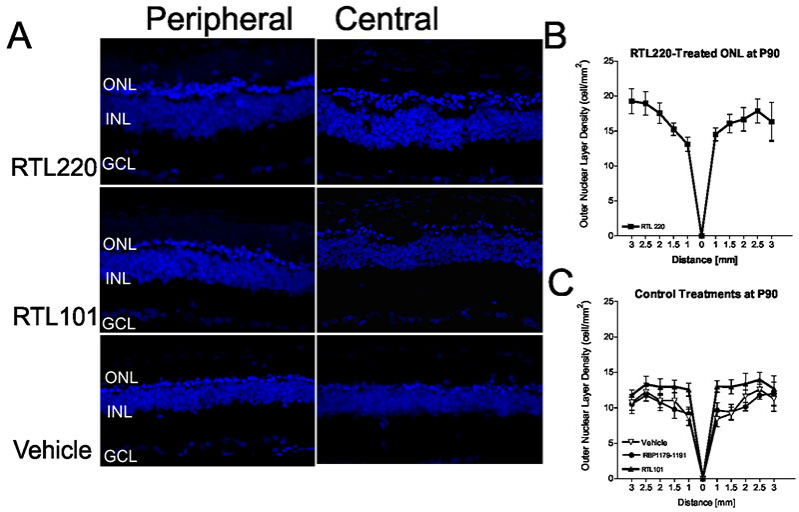
Preservation of photoreceptors nuclei was present in RTL220-treated retinas at P90 when treatment started at P30. Treatment of Royal College of Surgeons (RCS) rats with 20 μg RTL220 every other day preserved the outer nuclear layer (ONL) thickness at P90. **A**: Confocal images of retinas stained with diamidino-phenyl-indole (DAPI) for nuclei at P90 from RTL220-, RTL101-, and vehicle-treated RCS rats were taken from peripheral and central close to the optic nerve head region. **B**, **C**: Graphs represent measurements of the total ONL density in histological sections of each treatment group that were recorded at 0.5, 1.0, 1.5, 2.0, 2.5, and 3 mm from the optic disc region in RTL220-treated rats (**B**) and control rats (**C**). The error bars represent SD (n=6, one-way ANOVA p=0.0022). Abbreviations: INL - inner nuclear layer, GCL - ganglion cell layer.

In the next trial, the RTL220 treatment was administered in small daily doses (20 µg/day s.c.) starting at P30 and was performed until P90. The total RTL dose these rats received was reduced by half. RTL101 (“empty” molecule β1α1 without peptide) and IRBP peptide alone were used as control treatments to assess the long-term effect of administration of pathogenic peptide or vehicle (saline). At P90, we examined the number of remaining photoreceptors in the retina in the treatment and control groups by counting the total nuclei in the ONL. As shown in Figure 2, there was substantial preservation of photoreceptors in the RTL220-treated retinas. The total number of counted nuclei in the ONL from the RT220-treated retinas was significantly higher than in the retinas of the vehicle-treated controls (n=6, one-way ANOVA, p=0.0022). We observed three to four layers of the ONL in five out of six rats compared to a single layer in controls that was typical for dystrophic rats of this age (Figure 2A). RTL220 performed significantly better than RTL101 in slowing the degenerative process. RTL101 treatment showed one layer in the ONL similar to the vehicle-treated or IRBP peptide-treated controls. Together, these results show that RTL therapy protected photoreceptor demise by slowing the degenerative process.

In the RCS rat, visual function deteriorates with time as photoreceptors are lost [23]. To determine whether morphological preservation is correlated with visual improvement, we conducted optokinetic response studies under the photopic condition. We assessed the ability of the rats to track gratings of various widths that was quantified by the optokinetic reflex and measured as the spatial frequency threshold, or visual acuity [21]. We wanted to determine whether the RTL-treated rats compared to the age-matched controls had functional deficits that affected their visual acuity. The visual acuity measurements showed that rats treated with RTL220 performed significantly better than those with the RTL101 injection and the untreated age-matched controls (p<0.05) at P60 and P90 (Table 1). These findings correlated well with the morphological findings. In the untreated dystrophic RCS rats at P60, the visual acuity was 0.33±0.04 cycles/min and at P90 was 0.27±0.06 cycles/min. Table 1 summarizes our findings for the rats that received different doses of RTL220 and control RT101 starting at P21 and P30. The visual acuity measurement showed that rats treated with RTL220 performed significantly better than those with treated with RTL101 and the untreated age-matched controls (p<0.05) at P60 and P90. The difference between the RTL220 and RTL101 treatments at 100 and 200 μg RTL dose measured at the P90 time point was significant (p<0.05 on Dunnett’s multiple comparison test). The present results demonstrate that an optokinetic response system has great value for assessing vision in RTL-treated rats and is a useful initial indicator of visual function as has been repeatedly shown across RCS rat studies reporting on beneficial effects following various treatments [22-25]. In summary, RTL220 delivered at P21 or even P30 during the course of degeneration can preserve not only photoreceptor morphology but also the visual acuity in treated dystrophic rats.

**Table 1:** Visual acuity measured by optokinetic response (OKR) conducted on dystrophic rats that received RTL treatment.

**Treatment**	**Dose**	**Starting treatment**	P60*	P90*	Significance to untreated**
RTL220	200	P21		0.329±0.012	p<0.05, n=14
RTL220	200	P30		0.302±0.008	p<0.05, n=14
RTL220	100	P21		0.349±0.007	p<0.05, n=6
RTL220	50	P21	0.438±0.007		p<0.001, n=10
RTL220	20	P21	0.468±0.03		p<0.001, n=6
RTL101	200	P30		0.277±0.006	n=8
RTL101	100	P21		0.272±0.032	n=6
Untreated controls	0	n/a	0.33±0.04	0.27±0.06	n/a

*Mean±SEM including both eyes **p value was determined by one-way ANOVA.

### RTL220 prevents vascular denegeration

**Figure 3:**
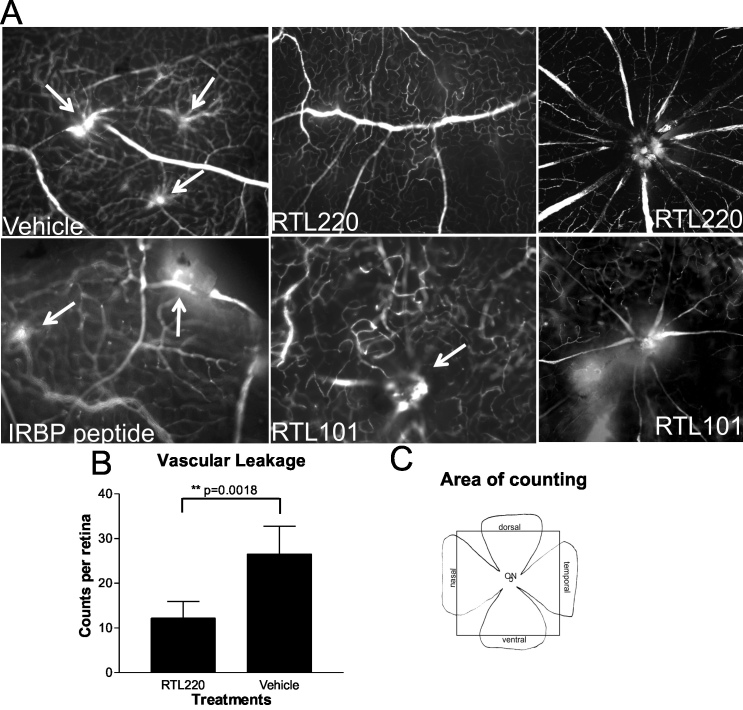
Typical angiography of retinal whole mounts is shown for different Royal College of Surgeons (RCS) rat treatment groups. **A**: Fluorescent micrographs represent RTL220, RTL101, IRBP1177–1191 peptide and vehicle-treated rats. The fluorescent pictures were taken from middle retinas around optic disc regions in **A** (RTL220 and RTL101), showing protective effects of RTL220 on the vasculature compared to the control rats presenting vascular leakage and abnormal vessels (arrows). **B**: Bar graph show the reduction in total counts of leaking vessels in RTL220-treated rats compared to the vehicle-treated rats (n=6, p=0.0018). **C**: Scheme of retinal flat mounts shows the area from which the leaking vessels were counted (box).

To examine whether RTL220 provides vascular protection at P90, retinal vessels were perfused with fluorescein isothiocyanate–dextran (fluorescent angiography), and the number of leaking retinal vessels was assessed in retinal whole mount preparations. Figure 3 shows representative fluorescent images of mid-retina and optic nerve areas in whole mounts from the control and RTL220-treated rats. In the RTL220-treated rats, the vascular leakage was greatly reduced, or no vascular leakage was present around the optic disc region. The summary of counts of all leaking vessels in the retinal whole mounts showed a significant reduction to about 50% after RTL220 therapy compared to the vehicle-treated rats (n=6, p=0.0018; Figure 3). In controls, vascular leakage was observed not only around the optic disc region but also in the vessels in the middle retina. RTL101 was not effective, suggesting that retinal and vascular protection with RTLs required a covalently-tethered peptide attached to the α1β2 polypeptide for maximum effectiveness.

**Figure 4:**
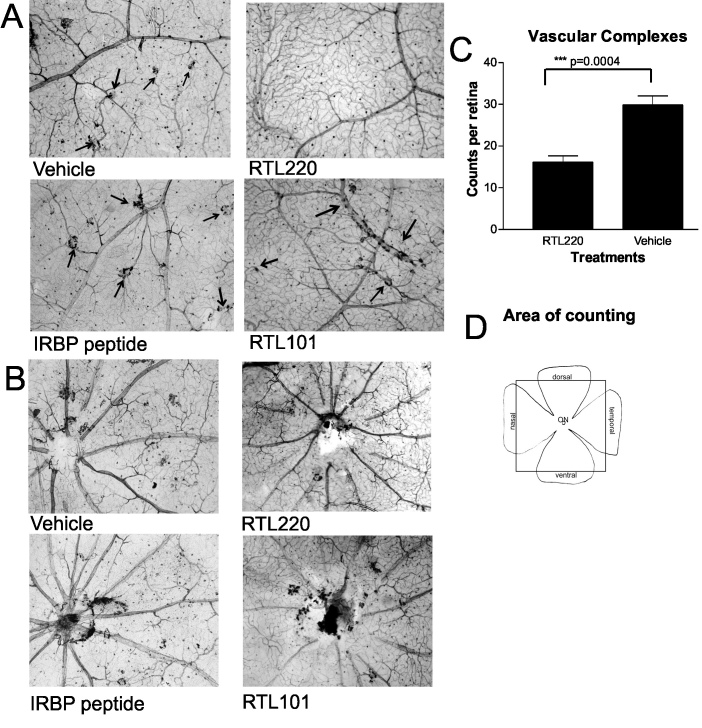
Representative vascular pathology is shown for retinal whole mounts stained with nicotinamide adenine dinucleotide phosphate (NADPH)-diaphorase to identify abnormal vessels associated with retinal pigment epithelium (RPE) cells (vascular complexes; arrows). **A**: Photomicrographs show the vascular complexes from middle part of the retina that were dramatically reduced in RTL220-treated rats compared to control rats. RTL101 was not effective and its effect was similar to vehicle- or plain interphotoreceptor retinoid binding protein (IRBP) peptide-treated RCS rats. **B**: Optic nerve head region view of RTL220 and control RTL101 treatment show the accumulation of pigmented cells around optic disc. **C**: Bar graph represents the overall reduction in vascular complexes in RTL220-treated retinas compared to vehicle-treated retinas (n=6, p=0.0004). **D**: Scheme of retinal flatmounts shows the area from which the abnormal vessels were counted (box).

After angiography, retinal whole mounts were stained with NADPH-diaphorase to evaluate the formation of vascular complexes composed of migrating pigmented cells. This method allowed the migrating RPE cells attached to pathological vessels to be identified. Figure 4 shows representative micrographs from RTL220-treated and control rats at P90 taken from areas around the optic nerve head region and mid-retina. The number of pathological vascular complexes was dramatically reduced in the RTL220-treated retinas, compared to the controls as follows: RTL220 < RTL101 < vehicle=IRBP_1177–1191_-treated rats (n=6/group). In particular, the RTL220-treated rats had greatly reduced vascular complexes around the optic disc and mid-retina, resembling non-dystrophic healthy retinas. In the controls, the vascular complexes were mainly located around the optic disc region but also spread around the middle and peripheral retinal vessels. The control group of rats that received the IRBP_1177–1191_ peptide, the same peptide that is part of RTL220, showed marked vascular changes similar to those seen in the vehicle-treated rats. Figure 4C shows the significant overall reduction in the total number of pathological vessels per retina in the RTL220-treated rat retinas compared to the controls (p=0.0018; n=6/group). Summarizing, therapy using RTL220 bearing the retina-specific IRBP_1177–1191_ peptide was effective not only in protecting photoreceptor cell morphology and visual function as shown with optokinetic response (OKR) but also in protecting the retinas of the RCS rats from vascular leakage and abnormal vascular formation even when the treatment was administered after the degenerative process had already begun (P21 and P30).

### Biodistribution studies support administration RTL220 for treatment

**Figure 5:**
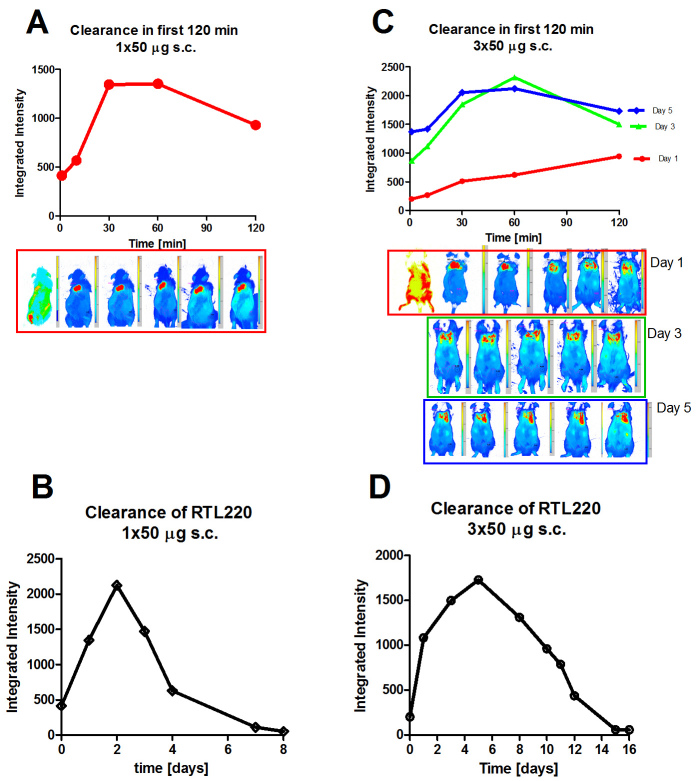
Clearance kinetics of RTL220 conjugated to fluorescent IRDye 800CW observed in the RCS rat. Representative data for 2 groups of rats (pictured) administrated with a single or triple dose of fluorescent RTL220 subcutaneously and monitored over time for its distribution and clearance. **A**: The rat received a single dose of 50 μg of RTL and was imaged at 0, 10, 30, 60, and 120 min. **B**: Daily imaging of the rat illustrate a slow dispersion of the fluorescent RTL from the injection site. **C**: The rat received 3 doses of the fluorescent RTL220 every other day and was imaged at 0, 10, 30, 60, and 120 min after each injection; **D**: Clearance of RTL220 after 3-dose treatment is shown over time to determine complete clearance.

The RTL represents a small protein of molecular weight (MW) about 22-kDa protein that eventually is metabolized. Blood clearance of RTLs has shown a relatively short blood half-life (20 min) after intravenous administration [26]. In our treatment studies, the RTL was delivered subcutaneously in repeated doses. We hypothesize that this administration route prolongs the exposure of the RTL by slower dispersion from the injection site and may yield long-lasting effects important in treating persisting retinal degeneration. Bioavailability studies were performed with a subcutaneous (s.c.) injection of fluorescent RTL220 conjugated to IRDye800CW dye and daily imaging of living rats. To illustrate the clearance of fluorescent RTL220 from the injection site we plotted integrated intensities obtained from individual rats shown in the picture that received a single or triple dose of fluorescent RTL (Figure 5). After a single s.c. administration of fluorescent RTL220, the intensity peaked at 30–60 min and sustained at the injection site for at least 2 days, suggesting a slow diffusion into the tissues. Figure 5A illustrates the first 120-min distribution of a single dose of 50 µg fluorescent RTL220. When rats received repeated doses (3×50 µg RTL220 every other day), the fluorescence at the injection sustained for about 12 days, was scattered around, and then almost completely cleared (approximately 90%) after 20 days (Figure 5C,D). A low variance was observed (SEM within 5%) between individual rats. Fluorescence in excised tissues collected at the end of experiment was assessed ex vivo. Low fluorescence was detected in some axillary and cervical lymph nodes, spleen, kidneys, and less in the liver, indicating that the RTL was cleared through the kidney without major accumulation in the liver. Histological staining of the kidneys did not show any pathological accumulation of the RTL compared to the normal untreated kidneys (not shown). In conclusion, we have demonstrated that RTL220 dispersed very slowly from the injection site, but the administration of multiple doses every other day was justified to maintain the same level of treatment.

### RTL220 suppresses immune responses during retinal degeneration

**Figure 6:**
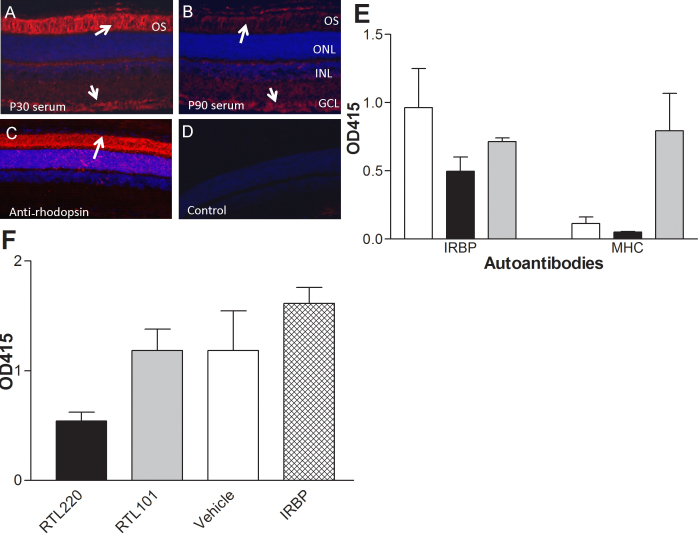
Immune responses in RCS rats.. **A-D**: Confocal images demonstrate labeling of non-dystrophic rat retina with autoimmune sera from untreated dystrophic RCS rats that was collected at P30 and P90 (dilution 1:20). **A**: Strong labeling of outer and inner segments was present at P30 (arrows), **B**: weaker immunolabeling of ganglion cell layer and outer segments in rat retina with serum antibodies collected at P90. **C**: Control staining of the non-dystrophic rat retina with anti-rhodopsin mAb (R2–12N, 1:1000) that strongly immunolabeled the outer and inner segments of photoreceptors. **D**: No labeling of retinal cells was observed in control immunolabeling without primary antibodies. **E**, **F**: RTL220 treatment inhibits autoantibody response against interphotoreceptor retinoid binding protein (IRBP). **E**: The bars represent autoantibody titers against IRBP1177–1191 and β1α1 MHC polypeptide (part of RTL) in serum from P60 rats (n=5) diluted 1:100 and measured by enzyme-linked immunosorbent assay (ELISA). Note that RTL220 decreased autoantibodies against IRBP1177–1191 peptide (black bars). Also rats produced antibodies against MHC when treated with RTL101 (β1α1 chain of MHC, gray bars). Vehicle treatment-white bar. **F**: RTL220-treated significantly reduced autoantibody levels against IRBP1177–1191 peptide (n=6, one-way ANOVA, p=0.0076) in sera from rats at P90 measured by ELISA at 1:100 dilutions. OS - outer segments, ONL - outer nuclear layer, INL - inner nuclear layer, GCL - ganglion cell layer, AAb - autoantibodies, MHC - major histocompatibility complex.

The basic action of RTL therapy is to disrupt T-cell responses against an antigenic peptide [19,27]. To test the hypothesis that RTL220 acts by suppressing antiretinal immune/autoimmune responses generated during photoreceptor cell death and protects the retina from degeneration, we evaluated T-cell and autoantibody responses after RTL therapy. Figure 6A-D shows immunofluorescent labeling of non-dystrophic retinas with RCS dystrophic serum autoantibodies. Serum collected at P30 strongly immunolabeled photoreceptor outer segments, but the labeling was weaker when dystrophic sera were collected later in the degenerative process. Because the rats received multiple doses of RTLs, there was also a concern that this treatment would generate an increase in T-cell responses and antibody titers against the IRBP peptide or epitopes present upon the MHC β1α1 polypeptide framework that would eventually neutralize the RTL220 action. However, the antibody titers against IRBP_1177–1191_ at the time points P60 and P90 were decreased (Figure 6E,F), except for the control animals that received IRBP_1177–1191_ peptide alone, which generated high antipeptide responses. Proliferative T-cell responses to IRBP_1177–1191_ were decreased to an undetectable level in rats treated with RTL220 at P60 and P90, suggesting that RTL220 effectively inhibited T-cell responses against IRBP peptide and other bystander responses (not shown). Collectively, these data indicate that RTL220 treatment may have a direct effect on autoreactive T cells and a partial effect on antibody production by B cells, suggesting that the beneficial outcome of RTL treatments in this disease model could be attributed to T-cell mediated inflammation but also to attenuation of B-cell activity during retinal degeneration.

**Figure 7:**
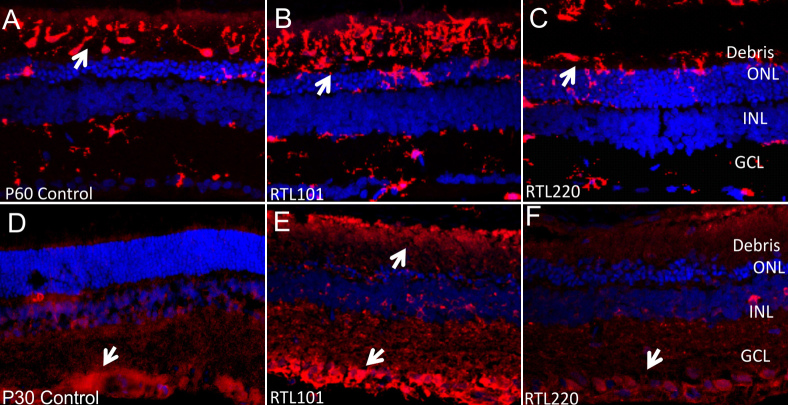
RTL220 protects the degenerating retina from microglia/macrophages (MG/mφ) activities. **A**-**C**: Representative confocal photomicrographs of the retina show the preservation of photoreceptor cells and reduction in MG/mφ after RTL220 treatment at P60. There is a significant reduction in MG/mφ migration and their presence in subretinal debris space in RTL220-treated retinas (**C**). MG/mφ were stained with lba-1 antibody (red) and nuclei with diamidino-phenyl-indole (DAPI; blue). **D**-**F**: Representative images show immunofluorescent staining of Royal College of Surgeons (RCS) rat retinas with anti- monocyte chemotactic protein-1 (MCP-1) antibodies (red) and nuclei with DAPI (blue). **D**: dystrophic retina at P30 shows MCP-1 expression around the ganglion cell layer; **E**: RTL101-treated retina. **F**: RTL220- treated retina shows a reduction in MCP-1 fluorescence; arrows indicate labeling of MCP-1 (red). Abbreviations: OS - outer segments, ONL - outer nuclear layer, INL - inner nuclear layer, GCL - ganglion cell layer.

The inflammatory activity can be also gauged by the presence of secondary effector immune cells such as microglia/macrophages (MG/mφ) in the retina that usually respond to pathological conditions involving immune system activation [28,29]]]]. Figure 7 shows immunofluorescent labeling of the RCS rat retina with anti-Iba1 antibodies (red) against MG/mφ markers, which are highly upregulated in activated MG/mφ. There was a striking decrease in the accumulating MG/mφ in the subretinal space in the RTL220-treated retinas measured at P60 where normally cellular debris was accrued during retinal degeneration in age-matched untreated rats. The same dose of control RTL101 did not inhibit the microglia activation, suggesting that RTL220 treatment prevents entry and migration of microglia into the subretinal space at the early stages of degeneration (Figure 7B). Microglia express their signaling molecule, monocyte chemoattractant protein (MCP-1 or CCL-2), which coincided with MG/mφ activation and their recruitment to the degenerating photoreceptor cell layer (Figure 7D-F). Initially MCP-1 immunofluorescence was observed within the ganglion cell layer at P30 and an increase with the migration of microglia during the degenerative process in the RCS rat retina. RTL220 considerably suppressed the production of MCP-1 compared to the RTL101-treated retinas, which correlated with the inhibited migration of the cells.

## Discussion

In this study, we showed that immunotherapy with RTL220 can effectively prevent progression of retinal degeneration in dystrophic RCS rats. The RCS rat is a classic model of recessively inherited retinal degeneration, in which the RPE fails to phagocytize shed outer segments, leading to photoreceptor cell death. In the untreated rat, there are changes in the outer segment appearance as early as 21 days of age, a time when the first early abnormalities appear in photoreceptors. By P60, the ONL is reduced from 10 to 12 cell layers to four to five cell layers. By 3 months of age, only a single row of photoreceptor nuclei remains, but the INL is unaffected and shows about five cell layers. The mechanism of retinal degeneration is complex and various factors play a role in the destructive process thus different preventive treatments of RCS rat retinal degeneration, including gene therapy [30,31], cell transplantation [32], or cell based therapy [22,33]by intraocular injection have been shown to slow degeneration. RCS rat photoreceptors have been also rescued by various growth and survival factors (basic fibroblast factor, ciliary neurotrophic factor), even when a thick layer of debris was present, suggesting that photoreceptor death is not due solely to the gene defect [30,34,35]. Here, we demonstrated that immunotherapy with RTL220 targeting chronic activation of immunity/autoimmunity limited retinal degeneration when delivered systemically by subcutaneous application to preserve fragile diseased eyes.

Most treatments seem to work when applied before pathology occurs. The goal was to evaluate whether this therapeutic approach can treat the ongoing retinal disease that human patients may experience. Therefore, we purposely designed our treatment regimens to examine whether RTL could stop or delay this degenerative process when delivered to dystrophic RCS rats at the first retinal abnormalities (P21) or when the degenerative process has already begun (P30). Our studies of the natural immune responses against retinal antigens in naïve RCS rats revealed a strong stimulation of anti-IRBP autoantibodies and T cells over the course of photoreceptor death but their activation trends show differences (Adamus, unpublished observation). The presence of serum autoantibodies correlated with early apoptosis of photoreceptors [36], suggesting that these autoantibodies were likely generated in response to the retinal antigens released from outer segments. The early defect in the interphotoreceptor matrix and the release of IRBP evident a week before the onset of photoreceptor apoptosis supports the notion that released antigenic material contributes to the initiation of natural immune responses [37,38]. We observed that autoantibodies against IRBP were continuously generated in RCS rats but a measurable activation of IRBP1177–1191-specific T cell responses developed after day 40, later in the course of degeneration (Adamus, unpublished observation). Perhaps early antigen-specific T cells were eliminated after activation, and only those that survived could be detected later. Recent studies using triple-knockout mice with a defect in the RPE similar to that in the RCS rats have also reported the presence of memory T cells and IRBP-specific CD4^+^ T cells in the mice [39]. The authors speculated that the persistent presence of such T cells makes triple-knockout mice more susceptible to the induction of uveitis than their non-dystrophic controls, proposing that rapid activation and reactivation of memory T cells play a role in pathogenicity [39]. The similarity in phenotypes between the two rodent models also suggests that the RPE phagocytic defect could contribute to retinal degeneration caused by the loss of function of MerTK in those rodents and perhaps in humans with MerTK defects [15,40]. RPE cells combined with cytokines play an active role in inflammatory and immunological responses in the retina [41,42]. It is not clear if changes in critical functions of RPE cells, including the maintenance of photoreceptor cell integrity by phagocytosing debris, epithelial transport of molecules, and metabolic waste, make those animals more susceptible to autoimmunity. However, such alterations in the RPE function may lead to the persistent availability of IRBP and other retinal autoantigens that stimulate and restimulate the antiretinal autoimmune responses until the photoreceptor cells finally die, and thus, immunotherapy with IRBP-specific RTL220 was particularly effective in suppressing retinal degeneration in RCS rats. Future studies in our laboratory using different models of retinal degeneration with different levels of immune system activation and gene defects will show whether RTLs are as effective as in RCS rats with malfunctioning RPE.

RTLs represent novel, bioengineered therapeutic molecules that do not contain the entire class II MHC molecules but consist of the membrane distal β1 and α1 domains with covalently linked antigenic peptides, including IRBP_1177–1191_ peptide [17,27,43,44]]]]. RTLs induce peptide-specific immunosuppression, and although in vitro studies have demonstrated that RTLs directly bind antigen-specific T-cell receptors and alter the signal transduction cascade of antigen-specific CD4^+^ T cells, the in vivo mechanism by which these molecules have such a profound ability to tolerize antigen-specific T cells appears to be far more complex [27,45]]]]. Recent evidence suggests that RTL therapy induced a tolerogenic state in CD11b^+^ antigen-presenting cells that results in tolerization of CD4^+^ T cells [46]]]]. Therefore, by inhibiting autoreactive T-cell responses, tissue-specific RTLs have been shown to reverse the clinical and histological signs in various acute experimental autoimmune disease models, including uveitis [16,17], encephalomyelitis, arthritis [45], and stroke [47] when applied with disease induction, at the priming phase, or at onset, whichever is the closest to a patient’s disorder. A human HLA-DR2 -derived RTL was recently used successfully in a phase I clinical trial for treating multiple sclerosis [48]]]]. Therapeutic activity directed against a single determinant (e.g., IRBP) as well as bystander effects against T cells reactive against other determinants (other IRBP determinants and retinal antigens) could explain how RTL220 might regulate multiple T-cell specificities that could contribute to protection [17,49]. Only RTLs with a covalently bound peptide, not empty RTL101 or pathogenic IRBP peptide alone, ameliorate retinal degeneration.

Microglial migration and accumulation in the debris zone were enhanced in the degenerating retina of the RCS rat and led to drastic photoreceptor degeneration and rapid loss of vision, suggesting that microglia contribute to retinal dystrophy [50,51]. The inhibition of microglial cell invasion into the photoreceptor cell layer promoted the survival of photoreceptor cells in the dystrophic retina [52]. Our findings support this observation and show that the immunotherapeutic effects of RTL220 helped to substantially reduce the number of migrating microglia into the ONL. The strong reduction of infiltrating MG/mφ by RTL220 treatment was related to the reduced expression of the MCP-1 chemokine required for entry into the CNS [53]. In addition, recent in vivo studies showed that RTLs preferentially bind with high affinity to antigen-presenting cells (APCs) such as macrophages, B cells, and dendritic cells, as well as platelets [46,54]]]]. RTLs might have a receptor on these APCs, and binding to such a receptor would induce an APC tolerization mechanism. Thus, RTLs could alter microglia migration and activation through the peptide-independent mechanism. The receptor has not been identified, but such studies are under investigation.

The loss of photoreceptors in retinal degeneration has been shown to have a profound effect on vascular development of the retina in RCS rats [55]. Vascular complexes, identified as abnormal vessels associated with clusters of RPE, were clearly evident by P90 in the control RCS retinas. The important beneficial finding from these studies was that RTL220 significantly also reduced vascular leakage and diminished formation of vascular complexes in contrast to vehicle-treated rats, which could be important in designing future treatments for RP and AMD. The mechanism is under investigation, but we speculate that RTL220 protected vasculature pathology via protecting the outer retina.

The major benefit of using RTL220 therapy to treat chronic retinal degeneration (RP, AMD) is that this therapy could eliminate multiple intraocular injections for drug delivery and complications associated with such injections. For now, we were able to delay and preserve some degree of visual function, rather than fully prevent the inevitable death of photoreceptors with genetically defective RPE in this model. Such treatment with RTLs might be more beneficial in human patients than dystrophic rats because disease progression in humans is much slower. The timing of treatment application and dose may improve beneficial effects, which we continue to investigate.

In summary, subcutaneous RTL220 delivery slows degenerative retinal disease, protects photoreceptors from destruction, promotes vascular survival, and protects visual acuity. The RTL has shown to be effective in treating acute (uveitis, optic neuritis) and now in the chronic form of eye disease leading to retinal degeneration. Future studies will fully explain the mechanism by which RTL therapy exerts its potent neuroprotective effects. In addition, RTL therapy could be a potentially attractive option for long-term treatment of chronic retinopathy following subcutaneous injection rather than being delivered into an already frail eye.
